# Genome-wide identification and functional characterization of RxLR effectors in *Phytophthora cinnamomi* infecting *Carya cathayensis* Sarg

**DOI:** 10.1080/21505594.2025.2590256

**Published:** 2025-11-14

**Authors:** Haonan Wang, Yaqi Fu, Xiaojie Peng, Zikun Li, Guiyong Cao, Feng Song, Lifeng Zhou, Yongjun Wang, Haiping Lin, Xudong Zhou

**Affiliations:** a National Key Laboratory for Development and Utilization of Forest Food Resources, Zhejiang A&F University, Hangzhou, China; b College of Foresty and Biotechnology, Zhejiang A&F University, Hangzhou, China; c College of Forestry and Landscape Architecture, Xinjiang Agricultural University, Urumqi, China

**Keywords:** Oomycete, *Phytophthora cinnamomi*, genome annotation, pathogenicity, RxLR effectors

## Abstract

*Phytophthora cinnamomi* is a globally distributed oomycete pathogen capable of infecting over 5,000 plant species, causing devastating root rot and stem canker diseases with significant agricultural and ecological impacts. Similar to other *Phytophthora* species, *P. cinnamomi* secretes RxLR effectors to suppress host immunity and facilitate infection. Here, we present a high-quality genome assembly of *P. cinnamomi* strain ST402 isolated from an economically important Chinese hickory (*Carya cathayensis* Sarg.), revealing 146 putative RxLR effectors. Transcriptomic profiling during the early infection stages (0–36 h post-inoculation) identified 66 differentially expressed RxLR effectors, with 4 highly induced candidates (PciRxLR1, PciRxLR21, PciRxLR57, and PciRxLR69) demonstrating cell death suppression activity against pathogen-associated molecular patterns (PAMPs) and promoting *Phytophthora* pathogenicity in *Nicotiana benthamiana*. Subcellular localization revealed distinct nuclear and cytoplasmic targeting patterns of these effectors. Our findings provide critical insights into the molecular mechanisms underlying *P. cinnamomi* virulence and lay the foundation for developing targeted control strategies against this destructive pathogen.

## Introduction

*Phytophthora cinnamomi* is one of the most devastating pathogens within the genus *Phytophthora*, with an exceptionally broad host range spanning over 5,000 plant species [1,2]. Its hosts encompass ecologically and economically vital species including eucalypts, pines, chestnut (*Castanea* spp.), oaks (*Quercus* spp.), avocado (*Persea americana*), macadamia (*Macadamia integrifolia*), and Chinese hickory (*Carya cathayensis* Sarg.) [3–5]. Comparative genomic analyses have revealed extensive repertoires of RxLR effector genes in notorious *Phytophthora* pathogens such as *Phytophthora sojae*, *P. ramorum*, and *P. infestans*, which are crucial for host immune suppression [6–8]. Notably, *P. cinnamomi* similarly employs RxLR effectors during infection, with a recent high-quality genome assembly predicting 181 putative RxLR effectors [9].

RxLR effectors are characterized by conserved N-terminal RxLR-dEER motifs that are essential for effector translocation into plant cells [10–12]. These virulence factors facilitate oomycete pathogenesis by subverting key components of the plant immune signaling pathways [13–15]. For example, the *P. sojae* effector PsAvh110 localizes to plant nucleus and disrupts the soybean GmLHP1-2/GmPHD6 complex to suppress host immunity [16]. Some other *P. sojae* effectors also target key proteins that play important roles in plant apoplast immunity [17,18]. *P. infestans* AVR8 targets desumoylating isopeptidase DeSI2, a positive regulator of plant defense [19], *Plasmopara viticola* RxLR50253 stabilizes the immune negative regulator VpBPA1 [20], *P. capsici* PcAvh1 acts as a key virulence determinant [21], and the RxLR effector PpE4 contributes to the *Phytophthora parasitica* infection [22]. The *Peronophythora litchii* RXLR effector PlAvh202 localizes to the plant cytoplasm and destabilizes a host ethylene biosynthesis enzyme to manipulate plant immunity [23]. Despite these advances, the molecular mechanisms underlying *P. cinnamomi* pathogenesis remain poorly characterized compared to those of other *Phytophthora* species.

Transcriptomic studies indicate that RxLR effectors with critical virulence functions are often highly expressed during the early infection stages [6]. Functional screening of these effectors typically reveals their capacity to suppress pattern-triggered immunity (PTI), such as programmed cell death (PCD) induced by BAX, INF1, or other Pathogen-Associated Molecular Patterns (PAMPs) [6]. Subsequent validation confirmed the essential roles of these upregulated effectors in promoting infection across multiple *Phytophthora* species [24–27]. Systematic identification and functional characterization of early-induced RxLR effectors in *P. cinnamomi* could elucidate the key virulence mechanisms and host interaction strategies.

Chinese hickory (*C. cathayensis* Sarg.), a high-value nut crop in Zhejiang Province of China, suffers severe yield losses owing to *P. cinnamomi*-induced dieback and root rot [5]. Building on our previous confirmation of *P. cinnamomi* ST402 as the causal agent [5], this study presents: a genome assembly of *P. cinnamomi* ST402 infecting *C. cathayensis* Sarg; prediction and characterization of 146 RxLR effectors; time-course transcriptomic profiling (0–36 h post-inoculation) of infected roots; functional validation of candidate effectors in suppressing PAMP-induced cell death and promoting pathogenicity.

Our findings provide critical insights into the effector biology of *P. cinnamomi* and establish a foundation for developing targeted control strategies against this economically significant pathogen.

## Results

### Genome assembly and annotation of P. cinnamomi ST402

The *P. cinnamomi* ST402 genome was sequenced using the PacBio long-read (35.62 Gb) and Illumina NovaSeq short-read (13.17 Gb) platforms. Initial estimation revealed a genome size of 85.6 Mb with 0.80% heterozygosity and 2.67% repetitive sequences (Figure S1). After a hybrid assembly and three rounds of polishing with Racon and Pilon, the final assembly comprised 1,681 scaffolds (N50 = 125.3 kb; longest scaffold = 1.416 Mb), yielding a total genome size of 127.90 Mb with 54.09% GC content (Table 1). BUSCO analysis using the Stramenopiles dataset demonstrated high assembly completeness, 93.7% complete genes (17.6% duplicated), 2.7% fragmented, and 3.6% missing (Table 1). The proportion of various results analyzed using BUSCO indicates the high quality of genome assembly in this study. It compares favorably to other available *P. cinnamomi* genome assemblies (Table S1).

**Table 1.** Characteristics of genome assembly of *Phytophthora cinnamomi.*

Assembly	
Assembled genome size (Mb)	127.90
GC Content (%)	54.09
No. of Scaffolds	1681
N50 length (bp)	125,285
BUSCO (%)	93.7
Number of predicted genes	22,619
Mean gene length (bp)	1,343
Number of secreted proteins	2,763
**Pathogenicity-related genes**	
RxLR effectors	149

Gene prediction via EVidenceModeler v1.1.1 identified 22,619 protein-coding genes, averaging 1,343 bp in length and occupying 23.65% of the genome. Gene Ontology (GO) annotation classified these genes into three functional categories: biological processes (27,451), molecular functions (8,087), and cellular components (12,912) (Figure S2). Notably, the observed gene count exceeded the initial predictions owing to the multifunctional annotation of individual genes, with predominant representation in cellular processes.

### Genome-wide prediction of P. cinnamomi RxLR effectors

**Figure 1.**
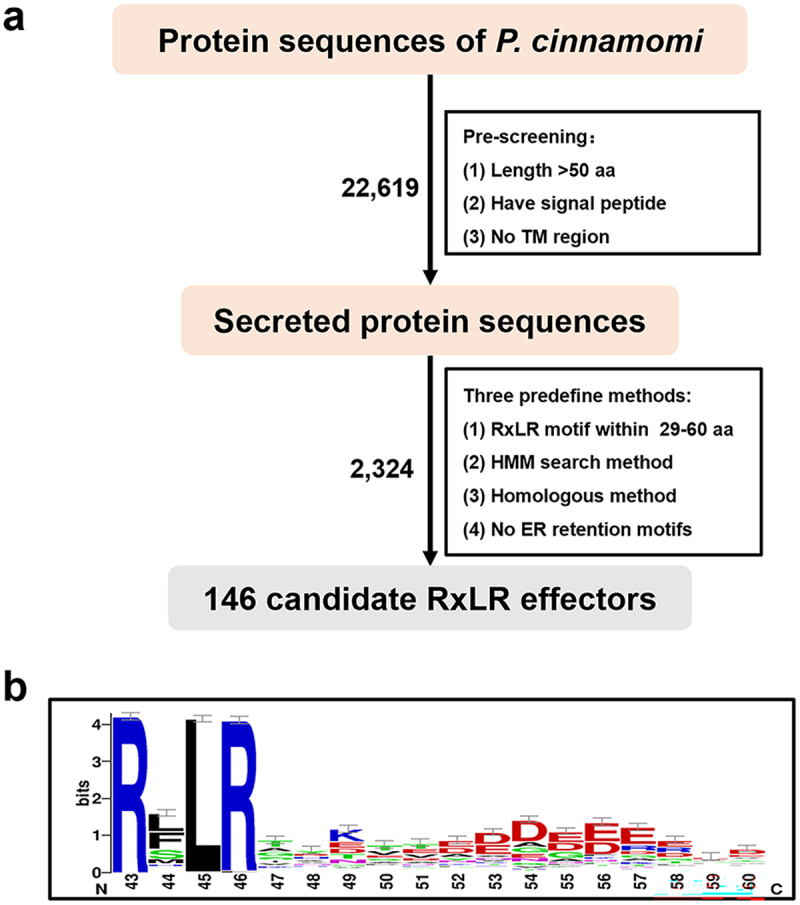
Identification of RxLR effectors in *P. cinnamomi*. (a) Flow chart of RxLR effectors prediction. Screen sequences with a length longer than 50 amino acids, containing signal peptides and without transmembrane domains from all encoded protein sequences, the secreted protein sequences were searched for predicting the RxLR-dEER motif using hidden Markov model, compare 38 homologous genes from *Phytophthora* to search for conservative RxLR motifs. (b) Weblogo analysis of *P. cinnamomi* RxLR effectors.

Given the importance of RxLR effectors in the infection process of *Phytophthora* plant pathogens, we performed a prediction of RxLR effectors in this assembled high-quality *P. cinnamomi* genome to facilitate subsequent functional analysis of their role in promoting pathogen infection. The *P. cinnamomi* genome was predicted to encode 22,619 protein-coding genes. Among them, 2763 proteins containing signal peptides were screened as secretory proteins using SignalP-6.0 (https://services.healthtech.dtu.dk/services/SignalP-6.0/). RxLR effectors manipulate plant immunity in the intracellular space of host plant cells; therefore, we predicted the transmembrane domain of these 2763 proteins with TMHMM, and removed 439 proteins with a transmembrane domain. Conserved RxLR motifs were identified by comparing 38 homologous genes of *P. infestance* using the Hidden Markov Model (HMM), and 116 protein sequences containing RxLR motifs were predicted. Protein-to-protein sequence alignment was performed using BLASTp alignment (E-value < 1e-5) with 1388 RxLR effectors used as references for *P. infestance*, *P. ramorum*, and *P. cinnamomi*, and 200 RxLR effectors were predicted [8]. Protein sequences were screened according to the method proposed by Win et al. [28], and those ranging from 29 to 60 amino acids that did not contain RxLR motifs were discarded. A total of 61 protein sequences containing RxLR motifs were identified. We predicted endoplasmic reticulum retention signals for all predicted effectors and removed 3 RxLR effectors containing endoplasmic reticulum retention signals. By integrating the RxLR effectors predicted using one of these methods, 146 candidate RxLR effectors were obtained, and detailed RxLR effector sequences and prediction criteria are provided in Table S2 (Figure 1(a), Table S2). WebLogo analysis confirmed the characteristic RxLR motif conservation across all candidates, and the RxLR-dEER domain used for mapping is provided in Table S3 (Figure 1(b), Table S3), with positional amino acid frequencies demonstrating strong conservation at RxLR-dEER core positions.

### Evolutionary dynamics of Phytophthora RxLR effectorss

**Figure 2.**
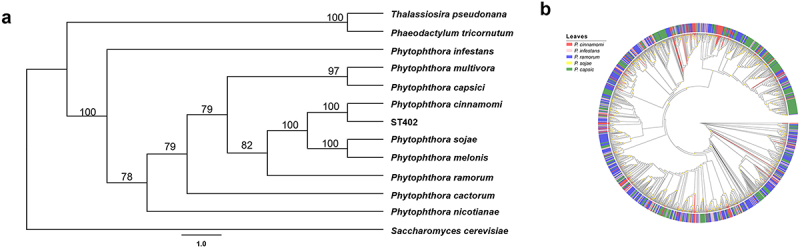
Evolution patterns of *Phytophthora* and *Phytophthora* RxLR effectors. (a) Phylogenetic analysing with *Saccharomyces cerevisiae* as the outgroup, using the 9 *Phytophthora* species, ST402 and 2 algae genomes information. The genome numbers of species can be found in accession numbers section. (b) Maximum-likelihood phylogenetic tree using RxLR-dEER motifs in RxLR effectors from five *Phytophthora* species. Yellow circles in the nodes indicate SH-aLRT values > 80%. Tree leaves are colored based on their respective species respectively. Node size is assigned in terms of bootstrap values. Tree leaves are colored based on their respective species. The branch containing the *P. cinnamomi* ST402 RxLR effectors was highlighted in red.

To elucidate the phylogenetic position of *P. cinnamomi* ST402, we reconstructed a maximum-likelihood tree using whole-genome sequences from representative *Phytophthora* species, with *Saccharomyces cerevisiae* as the outgroup (Figure 2(a)). The analysis revealed that ST402 forms a monophyletic clade with *P. cinnamomi*, exhibiting closer evolutionary relationships to *P. sojae* and *P. melonis* than to other congeners. Notably, the genetic distance between ST402 and the outgroup (*S. cerevisiae*) exceeded that observed between ST402 and two stramenopile algae species (*Phaeodactylum tricornutum* and *Thalassiosira pseudonana*), consistent with their shared stramenopile ancestry.

For RxLR-specific evolutionary analysis, we focused on functional domains by: truncating N-terminal signal peptides (essential for host cell entry) and removing variable C-terminal regions downstream of the conserved RxLR-dEER motif (associated with effector functions). This conserved evolutionary pattern aligns with the proposed “birth-and-death” model of effector gene evolution in oomycetes [29,30]. The resulting phylogenetic tree (Figure 2(b)), incorporated 146 *P. cinnamomi*, 208 *P. infestans*, 411 *P. capsici*, 282 *P. ramorum*, and 285 *P. sojae* RxLR effectors, demonstrated limited lineage-specific diversification. Table S4 presents the sequences of the *Phytophthora* RxLR effectors employed in constructing the phylogenetic tree. (Table S4). RxLR effectors from these species formed intermixed clusters without significant phylogenetic segregation (SH-aLRT > 80%), supporting their origin from a common ancestral repertoire prior to *Phytophthora* speciation [31]. We conducted an evolutionary analysis of effectors in different *P. cinnamomi* isolates and found that they are conserved within the species (Figure S5). The above results indicate that our predictions of the RxLR effectors in *P. cinnamomi* are reliable.

### Temporal profiling of P. cinnamomi RxLR effector expression during early infection

**Figure 3.**
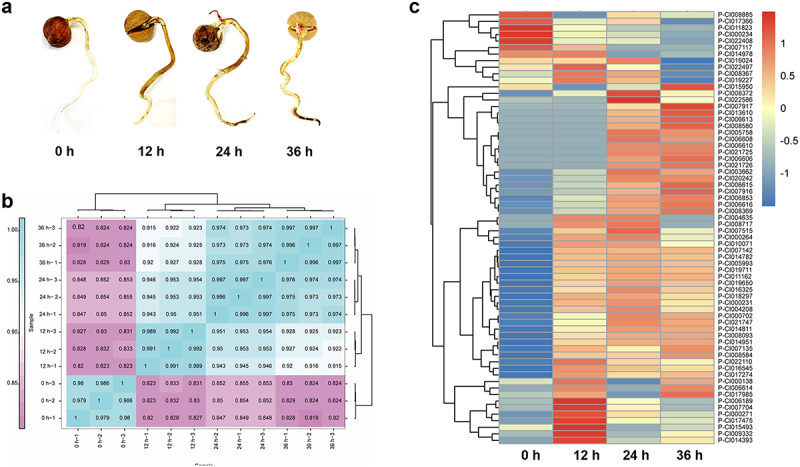
RxLR effectors expression levels after infection. (a) Germination of *C. cathayensis* Sarg. seeds in vermiculite, inoculate *P. cinnamomi* 3 weeks after *C. cathayensis* Sarg. seeds germination, collected samples 0 h, 12 h, 24 h and 36 h after inoculation using for rna seq. (b) Results of correlation analysis between samples at different stages of infection. (c) Differentially expressed RxLR effectors identified in different infection stages (0 h, 12 h, 24 h and 36 h after inoculation). The data used to draw the heatmap comes from Table S6. Heatmap was generated using the online tool (https://www.omicshare.com/tools/home/report/reportheatmap.html).

Effectors that play a crucial role during plant pathogen infection exhibit differential expression patterns in the early infection stage, which served as an important basis for our screening of key effectors. To identify the pivotal effectors among the numerous RxLR effectors in *P. cinnamomi*, we performed transcriptome sequencing on samples from different infection stages and screened for effectors that were differentially expressed during the process. To characterize RxLR effector dynamics during host colonization, *C. cathayensis* Sarg. roots were inoculated with *P. cinnamomi* ST402 zoospores, and samples were collected at 0 h, 12 h, 24 h, and 36 h post-inoculation (hpi) (Figure 3(a)). RNA-seq of these inoculated samples was conducted using the Illumina Novaseq 6000 platform, generating 150-bp paired-end reads. Clean reads were mapped to the *P. cinnamomi* ST402 genome using HISAT2 (v2.2.1) with default parameters [32], obtaining positional information on the reference genome or gene and sequence feature information unique to sequencing samples. Reads with Q30 scores < 90% were removed using Fastp, indicating high-quality RNA-seq data. Unique mapping rates of 88.64–92.23% and read pairing rates > 80%, indicating high-quality libraries (Table S5). Samples from different infection stages were analyzed, and the results showed that the correlation coefficients between samples from different treatment groups and control groups were close to 1 (Figure 3(b), Table S6), indicating that the results were highly repeatable and reliable. Principal component analysis (PCA) was conducted using the RNA-seq data. Different colored dots were clustered and distributed among different groups (0 h, 12 h, 24 h, 36 h) (Figure S3), indicating that the samples collected at different stages of infection were different. Differentially expressed genes between each infection and the non-infection stage were obtained (Figure S4a-c). The Venn diagram showed that the genes were differentially expressed at different infection stages (Figure S4d).

Expression-level clustering analysis was performed with the RxLR effectors detected at 0 h, 12 h, 24 h, and 36 h after *P. cinnamomi* infection to identify the important RxLR effectors during the infection process (Figure 3(c)). This was confirmed by the predicted RxLR effectors in the *P. cinnamomi* genome. A total of 66 RxLR effectors exhibiting staged expression patterns were detected. The detected RxLR effectors were assigned to 4 gene expression clusters using hierarchical clustering. Among the detected effectors, 6 effectors had higher expression levels at 0 h than at the other three infection stages, 18 effectors induced the highest expression levels at 12 h after infection, 24 effectors induced the highest expression levels at 24 h after infection, and 18 effectors induced the highest expression levels at 36 h of infection (Figure 3(c)). This staged expression program suggests the coordinated deployment of RxLR effectors during distinct phases of host colonization, with early-induced candidates potentially crucial for establishing infection.

### Functional validation of early-induced RxLR effectors in suppressing immune-related cell death

**Figure 4.**
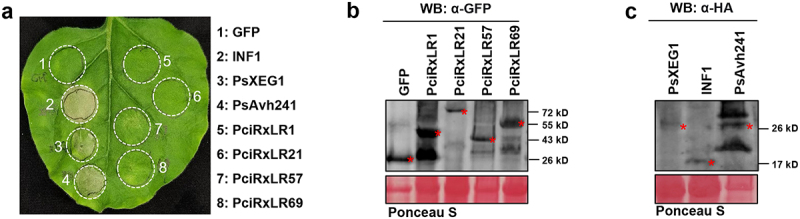
PciRxLR1, PciRxLR21, PciRxLR57 and PciRxLR69 cannot induce cell death. (a) The *P. cinnamomi* RxLR effectors could not induce plant immunity. Transiently expressing GFP as negative control and INF1, PsXEG1 and PsAvh241as positive controls, detecting whether the *P. cinnamomi* RxLR effectors could induce plant immunity. The picture was taken 72 h after agrobacterium mediated transiently expression. Similar phenotypes were observed in at least five independent experiments. (b) Expression of GFP and the *P. cinnamomi* RxLR effectors were confirmed by western blotting using anti-GFP antibody. (c) Expression of INF1, PsXEG1 and PsAvh241 were confirmed by western blotting using anti-HA antibody.

Through transcriptomic analysis, we identified four effectors that were upregulated during different stages of early infection and selected them as candidate key RxLR effectors for further study. The four effectors PciRxLR1 (P-CI006853.1), PciRxLR21 (P-CI022497.1), PciRxLR57 (P-CI011162.1), and PciRxLR69 (P-CI019650.1) with the highest expression levels at different infection stages, were cloned and tested for their ability to suppress plant cell death caused by PAMPs. The primers used to clone effectors were listed in Table S7 (Table S7). To assess their cell death-modulating activities, we performed *Agrobacterium tumefaciens*-mediated transient expression in *Nicotiana benthamiana* leaves, GFP serving as a negative control, and INF1, PsXEG1, and PsAvh241 as positive controls. Notably, none of the four effectors triggered visible cell death at 72 h post-infiltration (hpi) (Figure 4(a-c)), suggesting that they lack intrinsic cytotoxic activity.

**Figure 5.**
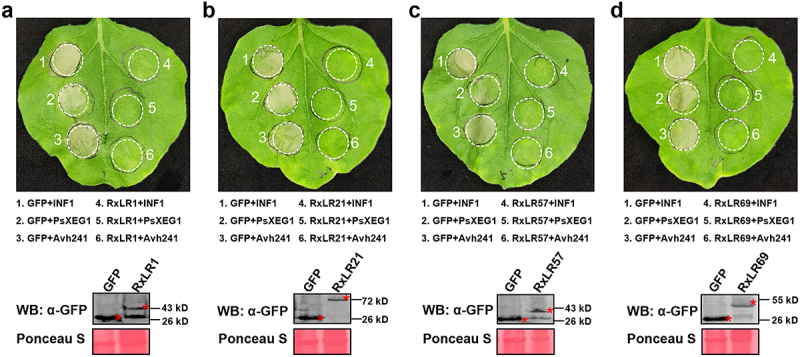
*P. cinnamomi* RxLR effectors highly induced in the early stages of infection could suppress the plant immunity. (a-d). Agrobacterium mediated transiently expressing GFP on left parts of *N. benthamiana* leaves and PciRxLR1, PciRxLR21, PciRxLR57 or PciRxLR69 on right parts of leaves. Overexpressing INF1, XEG1 and PsAvh241 48 h after GFP or RxLR effectors expressing. Pictures were taken 72 h after INF1, XEG1 and PsAvh241 expression. Similar phenotypes were observed in at least five independent experiments. Expression of GFP and the *P. cinnamomi* RxLR effectors were confirmed by western blotting using anti-GFP antibodies.

To clarify whether the *P. cinnamomi* RxLR effectors can suppress the cell death caused by PAMPs and effectors, we overexpressed the PAMPs (INF1, XEG1) or the effector that was reported to cause cell death (PsAvh241) 24 h after the injection of the above-mentioned four *P. cinnamomi* effectors or a negative control (GFP). The results show that all four effectors highly induced during the early infection stage can suppress cell death caused by INF1, XEG1, and PsAvh241 (Figure 5(a-d)). These findings demonstrated that *P. cinnamomi* temporally regulated RxLR effectors to sequentially disarm multiple layers of plant immunity during infection.

### RxLR effectors promote Phytophthora pathogenicity through distinct subcellular targeting

**Figure 6.**
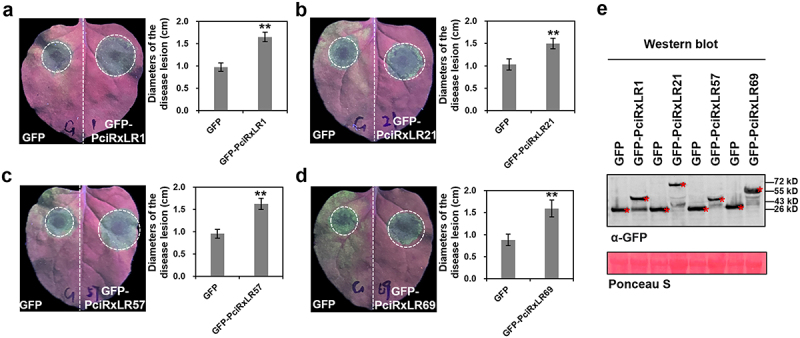
*P. cinnamomi* RxLR effectors highly induced in the early stages of infection could promote infection. (a-d) leaves transiently expressing GFP on left parts of *N. benthamiana* leaves and PciRxLR1, PciRxLR21, PciRxLR57 or PciRxLR69 on right parts of the leaves. Inoculating the *N. benthamiana* leaves with hyphae blocks (diameter 5 mm) of *P. capsici* 48 h after expressing. Observing the disease spots on the leaves and taking photos under uv light. Statistics of lesion length. Error bars represent the mean±s.D.(*n* = 10), and asterisks (**) denote significant differences (*p* < 0.01) between samples. The data used to build graphs are in Table S8. (e) Expression of GFP and the *P. cinnamomi* RxLR effectors were confirmed by western blotting using anti-GFP antibodies.

To explore the functions of these four highly induced *P. cinnamomi* effectors during the early infection stage, we overexpressed the four effectors and GFP on different sides of *N. benthamiana* leaves and inoculated *Phytophthora capsici* 48 h after injection. The results indicated that the *P. cinnamomi* effectors of PciRxLR1, PciRxLR21, PciRxLR57, and PciRxLR69 can promote *Phythphthora* infection (Figure 6, Table S8).

**Figure 7.**
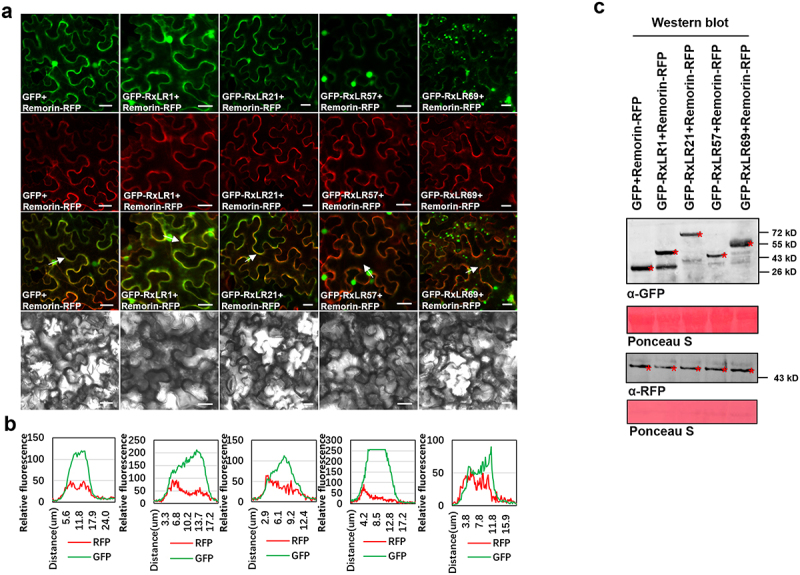
(a) Subcellular localization of PciRxLR1, PciRxLR21, PciRxLR57 and PciRxLR69. N-terminal GFP tagged PciRxLR1, PciRxLR21, PciRxLR57 or PciRxLR69 was expressed in *N. benthamiana*. Epidermal cells in the infiltrated tissues were investigated using confocal microscopy at 48 h post agroinfiltration. Scale bars, 20 μm. (b) Fluorescence analysis of GFP-PciRxLR1, GFP-PciRxLR21, GFP-PciRxLR57 and GFP-PciRxLR69/Remorin-RFP in membrane transects (white arrowheads). y axis, relative fluorescence intensity of GFP or rfp; x axis, transect length (μm). The data used to draw the line chart comes from Table S9. (c) Expression of GFP and the *P. cinnamomi* RxLR effectors were confirmed by western blotting using anti-GFP antibodies. Expression of remorin is confirmed by western blotting using anti-RFP antibodies.

We further overexpressed the effectors in the leaves of *N. benthamiana* to investigate their subcellular localization and explore their function after entering the plant host cells. Photographs taken using the confocal microscope illustrated that the effectors of PciRxLR1 and PciRxLR69 localized in the nucleus and cytoplasm, PciRxLR21 mainly in the nucleolus and PciRxLR57 mainly in the nucleus of plant cells. Table S9 presents statistics on the relative fluorescence intensity of green and red fluorescence (Figure 7, Table S9). These compartment-specific localization patterns suggest that divergent mechanisms of immune suppression-nuclear effectors may target transcriptional regulators, whereas cytoplasmic variants likely interfere with signaling cascades.

## Discussion

The genome of *P. cinnamomi* strain ST402 was assembled in this study, and the size is 127.90 Mb with a GC content of 54.09%. The genome of this oomycete species is highly heterozygous and contains numerous repetitive sequences. Several versions of the genome assembly of *P. cinnamomi* are available currently, and the number of scaffolds ranges from 1314 to 10,084, N50 from 10 to 264.5 Kb, and the estimated genome size from 53.69 to 77.97 Mb [9,33,34]. The *P. cinnamomi* genome included 1681 scaffolds with an N50 of 125.3 kb and an estimated genome size of 127.9 Mb. The quality of the generated genome assembly was not as high as that of Engelbrecht et al. with 133 scaffolds [9]. In this study, the genome size was increased owing to the use of short-read long sequencing technology. This better assembles repetitive regions that are likely to be missing in the other assemblies. Although nearly 100 oomycete genomes have been sequenced to date, only a few have been assembled into less than 1000 scaffolds. The optimal assembly of any publicly available *Phytophthora* species is the assembly of *P. sojae* containing 83 scaffolds, achieved through the sequencing of Fosmid and BAC libraries and primer stepping [35]. In recent years, researchers have attempted to use long-read sequencing technology to sequence the genomes of *Phytophthora* [36]. The results indicate that long-read-long sequencing technology has significant advantages in assembling the genome of oomycetes, but still poses significant challenges [36].

A total of 146 candidate RxLR effectors were predicted in the *P. cinnamomi* strain ST402 genome. This figure is much higher than the 68 RxLR effectors identified by McGowan et al. [37] but less than the 181 RxLR effectors identified by Engelbrecht et al. [10]. The process of predicting RxLR effectors is nearly as same as Engelbrecht et al. [10]. We speculate that there are two main reasons for the differences in the predicted number of RxLR effectors. First, *P. cinnamomi* we used for genome sequencing have different strains, and there may be differences in their genomic sequences. Secondly, the quality of our genome assembly is not as high as theirs, the BUSCO analysis result was 93.7%, which is also significantly lower than that reported by Engelbrecht et al. (97.5%), indicating a relatively lower completeness of the genome assembly in this study. The reason for this assembly discrepancy may be that the increase in genome size is due to the use of short-read sequencing technology, which better assembled repetitive regions that were likely missing in other assemblies. The differences between *P. cinnamomi* strains and the quality of genome sequencing have to some extent affected the subsequent prediction of RxLR effectors, resulting in differences in the number of RxLR effectors prediction. RxLR effectors are important virulence factors that suppress host immune responses to promote *Phytophthora* infection. RxLR effectors prediction is based on three different criteria: the Win method, Regex method, and Hidden Markov Model (HMM). Among the predicted 146 RxLR effectors, 42 satisfied all three criteria, 61 satisfied two criteria, and 46 satisfied only one criterion. Combining transcriptomes at different infection stages, 66 RxLR effectors were detected for gene-expression level changes, showing a staged expression pattern. Among these, 60 RxLR effectors had gene expression levels higher than 0 h during the infection stages. We speculate that the RxLR effectors induced for high expression during the infection stage play a role during infection by *P. cinnamomi*.

Although we predicted 146 RxLR effectors from the ST402 genome, only 66 differentially expressed effectors were detected in transcriptome sequencing. Gene expression changes must cross a certain threshold (in both statistical significance and fold-change) to be detected. The total number of reads in an RNA-Seq experiment (sequencing depth) determines the ability to detect lowly expressed genes. With insufficient depth, transcripts that are expressed at low levels or change subtly will be missed because there is not enough data to statistically confirm the change. The analysis relies on the ST402 strain genome; unfortunately, we cannot assemble this genome with the same high quality as the *P. sojae* genome, and the quality of the genome may also affect the results of transcriptome analysis. The samples we used for transcriptome sequencing were mixed samples of *C. cathayensis* infected with *P. cinnamomi*. Therefore, the proportion of *P. cinnamomi* tissue in the samples was relatively small, which also increased the difficulty of the detection of *P. cinnamomi* genes during transcriptome sequencing. It is actually quite common in transcriptome analysis, for example, there are only 41 *P. cinnamomi* RxLR effectors that were detected and differentially expressed among 181 predicted RxLR effectors in the research of Engelbrecht et al. [10].

PAMPs, such as INF1 and PsXEG1, can induce plant immunity [38,39], and Avh241 can induce cell death in *N. benthamiana* [6]. PsXEG1, INF1, and PsAvh241 were selected to test whether the *P. cinnamomi* RxLR effectors that induced high expression during the early stages of infection could suppress the cell death caused by plant immunity. We cloned the four RxLR effectors PciRxLR1, PciRxLR21, PciRxLR57, and PciRxLR69, which had the highest expression levels at four different infection stages and found they could suppress the cell death caused by PAMPs. Further investigation disclosed that the four effectors also promoted the infection of *Phytophthora*. Our study showed that *P. cinnamomi* RxLR effectors contribute to pathogen infection, studying the RxLR effectors of *P. cinnamomi* facilitates the elucidation of its pathogenic mechanisms.

The subcellular localization observation results of RxLR effectors show that PciRxLR1 and PciRxLR69 are localized in the nucleus and cytoplasm, PciRxLR21 is mainly in the nucleolus, and PciRxLR57 is mainly in the nucleus of plant cells (Figure 7). Effectors targeting plant cells typically regulate plant immune responses by interfering with the transcription of resistance-related proteins within plant cells. The nuclear effector PsAvh110 specifically targets GmLHP1-2. Thereby disrupting the formation of the GmLHP1-2/GmPHD6 complex and thus suppressing its transcriptional activity [16]. PsAvh23 suppresses H3K9 acetylation mediated by the ADA2/GCN5 module, which enhances plant susceptibility. The misregulation of defense-related genes upon PsAvh23 expression or GmADA2/GmGCN5 silencing further confirms this disruptive effect [40]. The effector located in the cytoplasm of plants inhibits plant immunity by infecting organelles within the cytoplasm. The soybean pathogen *P. sojae* employs an essential effector protein, PsAvh262, to stabilize endoplasmic reticulum (ER)-luminal binding immunoglobulin proteins (BiPs). These BiPs function as negative regulators of plant immunity against Phytophthora. Through the stabilization of BiPs, PsAvh262 suppresses ER stress-induced cell death, thereby promoting infection by Phytophthora [41]. Therefore, we hypothesize that *P. cinnamomi* effectors also modulate plant immunity through a similar mechanism. In future studies, we will focus on the relationship between the localization of these effectors and their virulence functions.

In summary, we sequenced and assembled the genome of the *P. cinnamomi* strain ST402 and predicted the RxLR effectors. We performed transcriptome sequencing with samples during the process of different infection stages and selected the RxLR effectors that were differentially expressed. An experiment conducted using *N. benthamiana* showed that these RxLR effectors contributed to *Phytophthora* infection. The knowledge gained here contributes to the understanding of the pathogenic and pathogen–host interaction mechanisms of *P. cinnamomi*, a notorious agricultural and forestry pathogen worldwide.

## Materials and methods

### Construction of genome sequencing library

*P. cinnamomi* strain ST402 was inoculated into V8 liquid medium and cultured for 2 d. Mycelia were collected for DNA extraction, and sequencing was conducted by Shanghai Biozeron Biotechnology Co., Ltd.

Illumina TruSeq™ Nano DNA Sample Prep Kit1: Covaris M220 ultrasonic disruptor was used to break the DNA sample to the target size, usually 300–500 bp. Perform a 3’-end repair and dephosphorylation treatment on the interrupted DNA fragment, followed by 3’-end poly (A) treatment. PCR was used for eight cycles of enrichment amplification to amplify DNA fragments with index tags. The target fragment of the expected size was recovered in 2% ultrapure agarose gel. Bridge PCR amplification was performed on an Illumina cBot vector to generate a large number of DNA clusters on the solid-phase carrier. The prepared DNA clusters were loaded onto the Illumina NovaSeq sequencing platform and perform double-ended sequencing was performed, with each end sequencing length of 150 bp.

PacBio: The G-tubes method was used to break genomic DNA into long fragments of 15–20 kb. Perform end repair on the interrupted DNA to ensure no damage or sticky ends and connect both ends to a closed loop structure. After annealing, the open circular structure of the single-stranded circular DNA template allows single-stranded DNA to fold and bind to DNA polymerase fixed at the bottom of zero-mode waveguides (ZMW) for fixation. After template loading was completed, sequencing was performed on the PacBio Sequel II sequencing platform.

### Genome assembly

The software MaSuRCA v4.0.8 (http://www.genome.umd.edu/masurca.html) Hybrid assembly of Illumina reads and PacBio long reads was applied, and it combines the advantages of de Bruijn and Overlap Layout Consensus. The assembly process involves the initial assembly of short reads to generate super reads and comparing the super reads obtained in the previous step with the extended long reads to obtain mega reads. We assemble mega reads using Celera Assembler and remove redundancy to obtain the assembly results.

### Genome annotation

The reference genome was trained using AUGUSTUS v3.2.3 software (http://bioinf.uni-greifswald.de/augustus/) for de novo gene prediction. Homological alignment prediction was performed using the protein sequences of the reference genome. Align the protein sequence to the sample genome sequence, remove redundancy, and then run Genewise v2.4.1 (https://www.ebi.ac.uk/seqdb/confluence/display/THD/GeneWise) to perform a precise comparison to determine the gene coding region and intron region. Use TopHat v2.1.1 software (http://ccb.jhu.edu/software/tophat/index.shtml) to align transcriptome data to the genome sequence and Trinity v2.11.0 software (https://github.com/trinityrnaseq/trinityrnaseq/releases) to obtain transcripts. EVidenceModeler v1.1.1 (http://evidencemodeler.github.io/) to integrate the above gene sets to obtain the encoded genes of the sample genome. Use BUSCO (https://busco.ezlab.org/) to evaluate the integrity of the gene set.

### Candidate RxLR effectors prediction

SignalP-6.0 (https://services.healthtech.dtu.dk/services/SignalP-6.0/) was used to perform N-terminal signal peptide prediction and to retain candidate protein sequences containing signal peptides. Predicted transmembrane domains for candidate proteins containing SPs were achieved using TMHMM-2.0 (https://services.healthtech.dtu.dk/services/TMHMM-2.0/) [42]. A combination of the HMMsearch method, hidden Markov model, and software package HMMer 2.3.2 was used to query a large set of sequences to detect RxLR motifs.

### RNA sequencing

RNA sequencing was performed by Shanghai OE Biotech Co., Ltd. The constructed RNA-seq library was loaded onto the Illumina Novaseq 6000 system, the read length was set to 150 bases, and double-ended sequencing was used. After sequencing is completed, raw read data in FASTQ format are generated. FastP software was used to remove adapters, filter low-quality bases, and eliminate low-quality reads from the raw reads. The resulting clean reads were used for subsequent data analysis.

### Differential expression gene analysis

Differential expression gene analysis was performed using DESeq2 v1.40.2 for three computational tasks: Normalization of raw read counts across samples through median-of-ratios scaling, generating BaseMean values as normalized expression indices; Quantification of differential expression through log2 fold change calculations; Statistical significance assessment via negative binomial generalized linear models with Wald test [43]. Differentially expressed protein-coding genes were identified by applying dual thresholds of |log2 fold change| ≥ 1 (equivalent to 2-fold differential expression) and Benjamini-Hochberg adjusted p-value < 0.05. For samples lacking biological replicates, the DESeq analytical framework was implemented via mean-variance shrinkage estimation (parameter settings: fitType=“mean,” betaPrior = FALSE) to enhance statistical robustness [44], with independent filtering enabled to maximize detection power while controlling false discovery rate.

### Phylogenetic analysis

The complete genome sequences were downloaded from NCBI (https://www.ncbi.nlm.nih.gov/) for phylogenetic analysis. This included sequences of *P. cinnamomi* strain ST402, *P. cinnamomi*, *P. cactorum*, *P. sojae*, *P. capsici*, *P. infestans*, *P. melonis*, *P. multivora*, *P. multivora*, *P. nicotianae*, *P. ramorum*, *P. tricornutum*, and *T. pseudonana*, with *S. cerevisiae* as the outgroup. For phylogenetic reconstruction, the signal peptide regions (SPs) and variable C-terminal domains downstream of the conserved dEER motif were excised from RxLR effector sequences. Processed amino acid sequences were initially aligned using MUSCLE v5.1 with default parameters, followed by manual curation to optimize positional homology. Maximum likelihood phylogenies were inferred through IQ-TREE 2.2.2.7 under the VT+F+R6 evolutionary model, which was identified as optimal through Bayesian Information Criterion (BIC) evaluation using the integrated ModelFinder algorithm. Node support was assessed via 1,000 ultrafast bootstrap replicates (−bb 1000). Final tree visualization and annotation were performed using EvolView v3 web platform with circular layout configuration [31].

### Plant and pathogen materials

*C. cathayensis* Sarg. roots used for infection and RNA-seq were grown in greenhouses at 25°C for 7 weeks, and the seeds of *C. cathayensis* Sarg. were bought from Hangzhou Tianzeshan Forestry Technology Co., Ltd. *N. benthamiana* first collected by Benjamin Bynoe and housed at the Royal Botanic Gardens, Kew [45]. *N. benthamiana* plants used for overexpression and infection were grown in greenhouses at 25°C for 5 weeks, and the seeds of *N. benthamiana* were given by Professor Wang Yuanchao from Nanjing Agricultural University. *P. cinnamomi* strain ST402 was provided by Yongjun Wang, separated from *C. cathayensis* Sarg. *P. cinnamomi* and *P. capsici* were cultured in 10% V8 medium at 25°C in the dark for 3 d.

### Gene clone and plasmid construction

*PciRxLR1*, *PciRxLR21*, *PciRxLR57*, and *PciRxLR69* were cloned from *P. cinnamomi* (ST402) cDNA, and the primers used for gene cloning are shown in Table S5. The fragments were fused to vectors by homologous recombination using a ClonExpress Ultra One Step Cloning Kit (Vazyme Biotech Co., Ltd.).

### Agrobacterium-mediated transient expression in N. benthamiana

The *A. tumefaciens* strain GV3101 was grown in a Luria Bertani (LB) medium at 28°C for 24 h. Centrifugal collected *A. tumefaciens* and resuspended in an infiltration buffer (10 mM MgCl_2_, 200 lM acetosyringone, 1 mM MES, pH 5.6). Adjust the concentration of *A. tumefaciens* to OD_600_ = 0.6 for the infection of *N. benthamiana*.

### Phytophthora infection experiments in N. benthamiana

For *P. capsici* infection assays on agroinfiltrated *N. benthamiana* leaves, leaf samples were harvested at 48 h post-infiltration with *A. tumefaciens* and subsequently inoculated with 10% V8 juice agar discs (5 mm diameter) containing fresh *P. capsici* LT263 mycelial cultures. Disease progression was assessed after 36–48 h of inoculation by measuring lesion diameters and documenting symptom development through photography under UV illumination.

### Subcellular localization observation

*N. benthamiana* leaves were cut and placed on a glass slide with distilled water and analyzed using the LSM 880 laser scanning microscope (Carl Zeiss, Germany) with a 10 × objective lens. Green fluorescence was observed by excitation at 488 nm.

### Western blotting

Following the SDS-PAGE separation (Prestained Color Protein Marker # P0068, Beyotime), proteins were electrophoretically transferred onto PVDF membranes. The membranes were subsequently blocked with 5% (w/v) nonfat milk prepared in a PBST buffer (phosphate-buffered saline containing 0.1% Tween-20) under constant agitation (60 rpm) at room temperature for 30 minutes. Primary antibodies diluted in a blocking buffer were then applied for target protein detection. Antibodies used were: anti-GFP (1:5000, #M20004, Abmart), anti-HA (1:5000, #M20003, Abmart), and anti-RFP (1:5000, # MD59004, Abmart). After the addition of the antibodies, the membranes were incubated at room temperature for 1.5 h. Washed the membranes 3 times with PBST buffer, and incubated with the goat anti-mouse (Odyssey, no. 926 – 32,210, Li-Cor) for 30 min. Figure S6 shows the uncropped images of all Western blots mentioned in the manuscript.

## Accession numbers

The genome data used for Phylogenetic analysis were downloaded from NCBI (https://www.ncbi.nlm.nih.gov/): *Saccharomyces cerevisiae* (GCA_000146045.2), *P. cinnamomi* (GCA_018691715.1), *P. cactorum* (GCA_016864655.1), *P. sojae* (GCA_000149755.2), *P. capsici* (GCA_030324255.1), *P. infestans* (GCA_000142945.1), *P. melonis* (GCA_024679155.1), *P. multivora* (GCA_001314345.1), *P. multivora* (GCA_003328465.1), *P. nicotianae* (GCA_003328465.1), *P. ramorum* (GCA_020800215.1), *P. tricornutum* (GCA_000150955.2), *T. pseudonana* (GCA_000149405.2).

## Supplementary Material

Table S6.xlsx

Figure S3.tif

Figure S5.tif

Table S4.xlsx

Table S9.xlsx

Figure S1.tif

Table S3.xlsx

Table S7.xlsx

Figure S4.tif

Table S5.xlsx

Figure S2.tif

Table S1.xlsx

Figure S6.tif

Table S8.xlsx

Supplementary figures （clean version）.docx

Table S2.xlsx

## Data Availability

The genomic data were deposited in GenBank under the accession number PRJNA1185894. Raw sequence reads were deposited in the NCBI SRA database under the accession number PRJNA1178270. Supplementary tables and figures have been uploaded to the Figshare database (https://doi.org/10.6084/m9.figshare.28844555) [46]. The authors confirm that the data supporting the results of this study are available in the article and its supplementary materials.
