# Supplementary figures and images for: Genome-wide identification and functional characterization of RxLR effectors in Phytophthora cinnamomi infecting Carya cathayensis Sarg
Source: Virulence. 2025 Nov 14;16(1):2590256. doi: 10.1080/21505594.2025.2590256 (PMC12629336; doi:10.1080/21505594.2025.2590256)

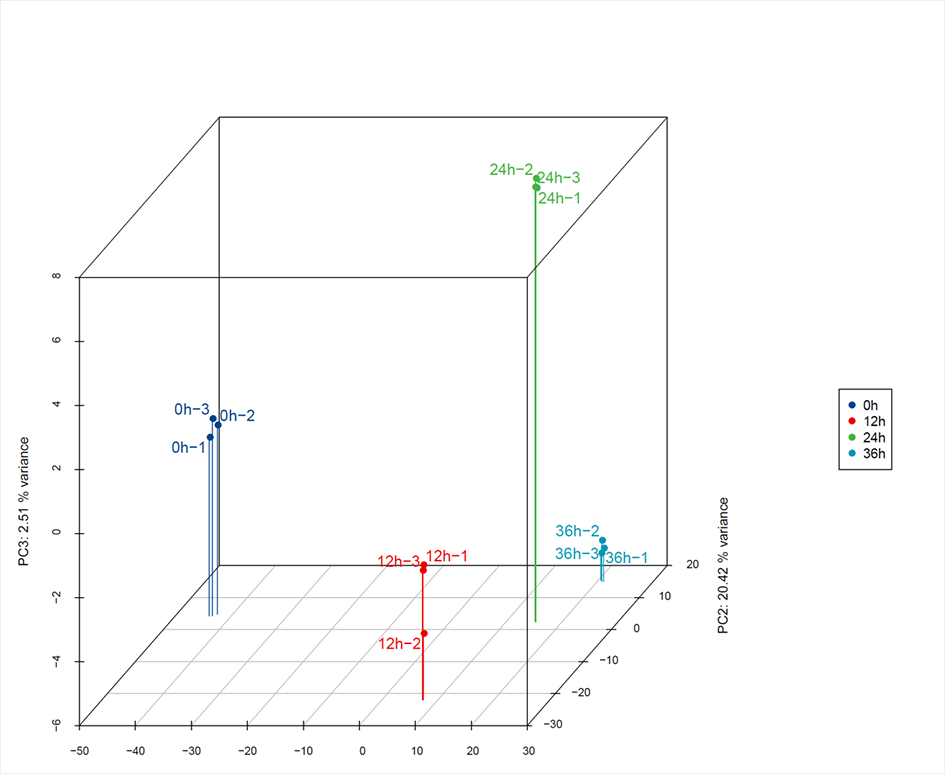

Supplement: Figure S3.tif [file KVIR_A_2590256_SM8840.tif]

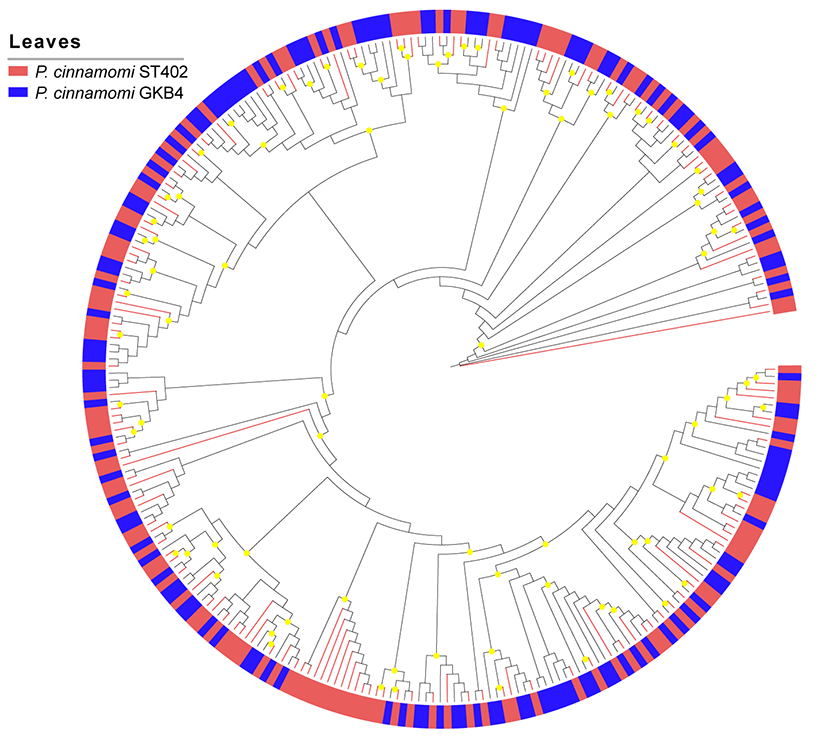

Supplement: Figure S5.tif [file KVIR_A_2590256_SM8839.tif]

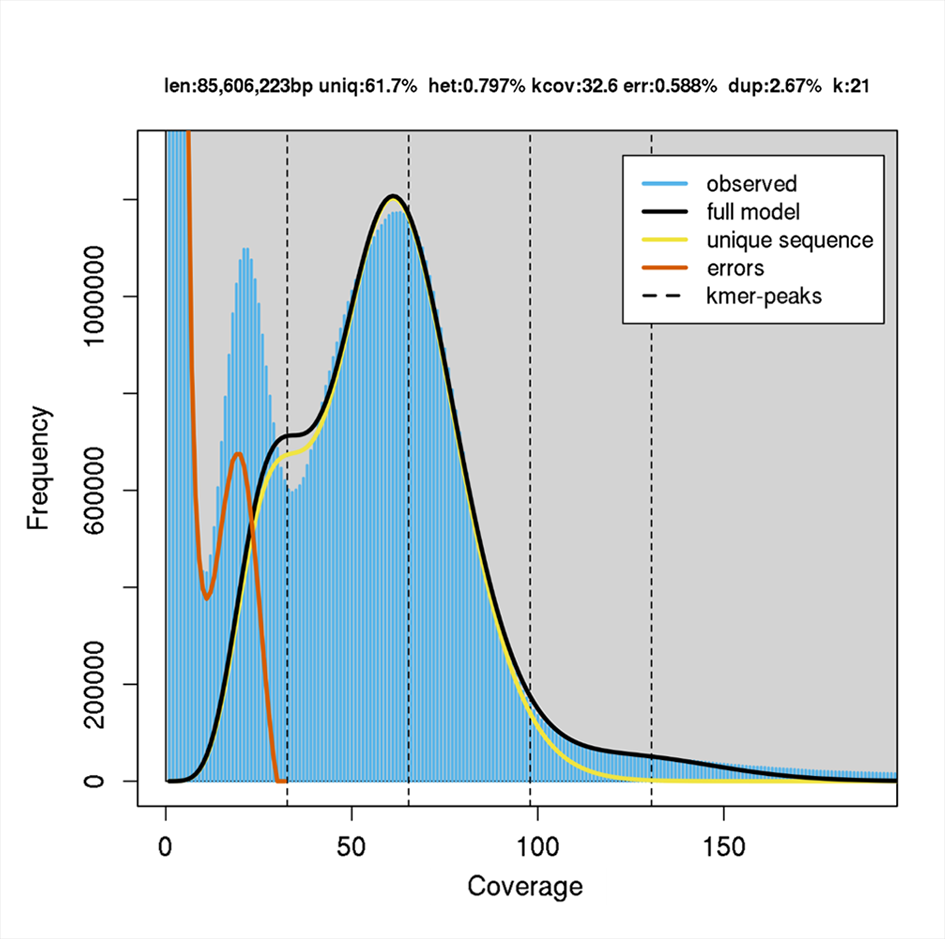

Supplement: Figure S1.tif [file KVIR_A_2590256_SM8836.tif]

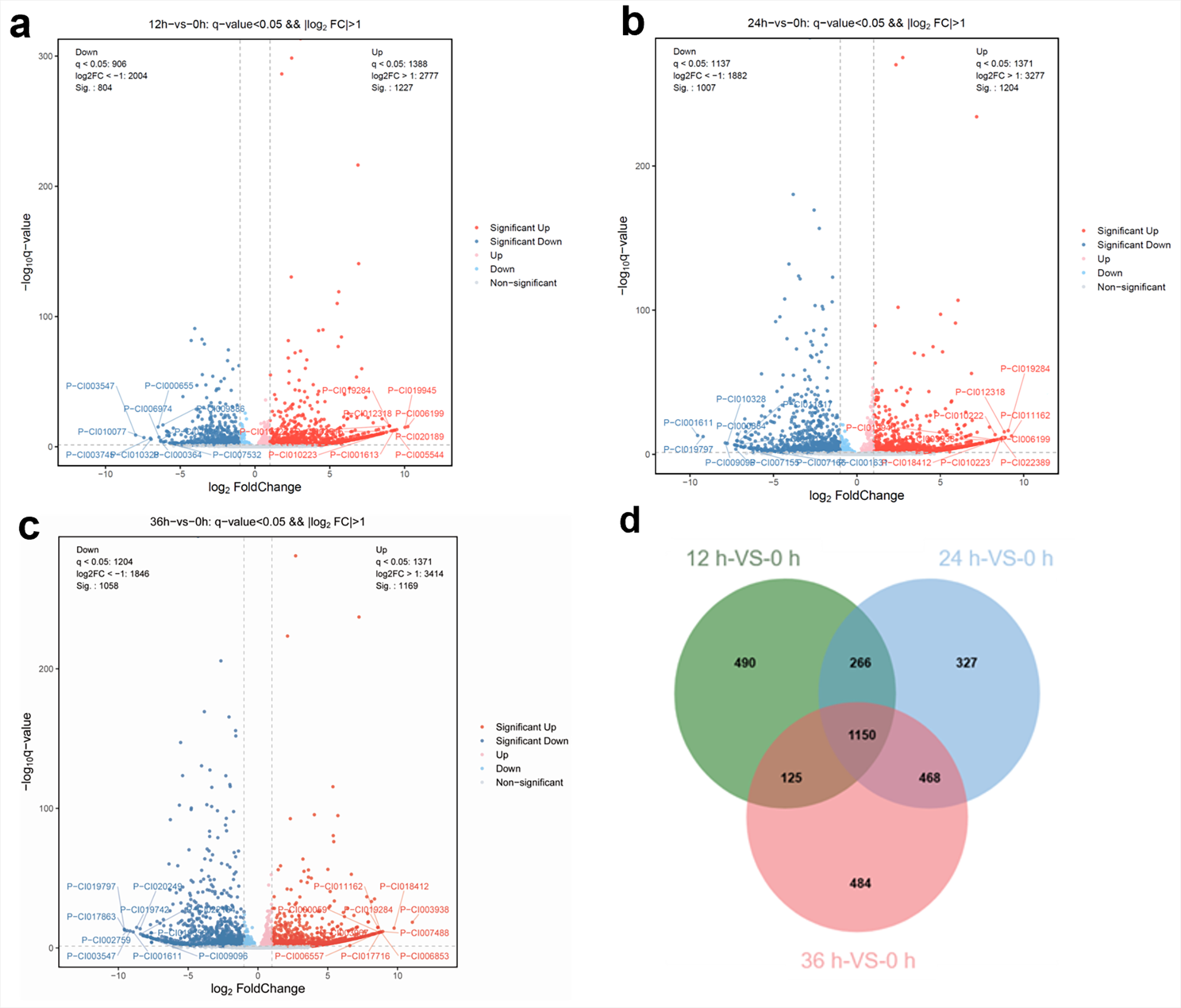

Supplement: Figure S4.tif [file KVIR_A_2590256_SM8833.tif]

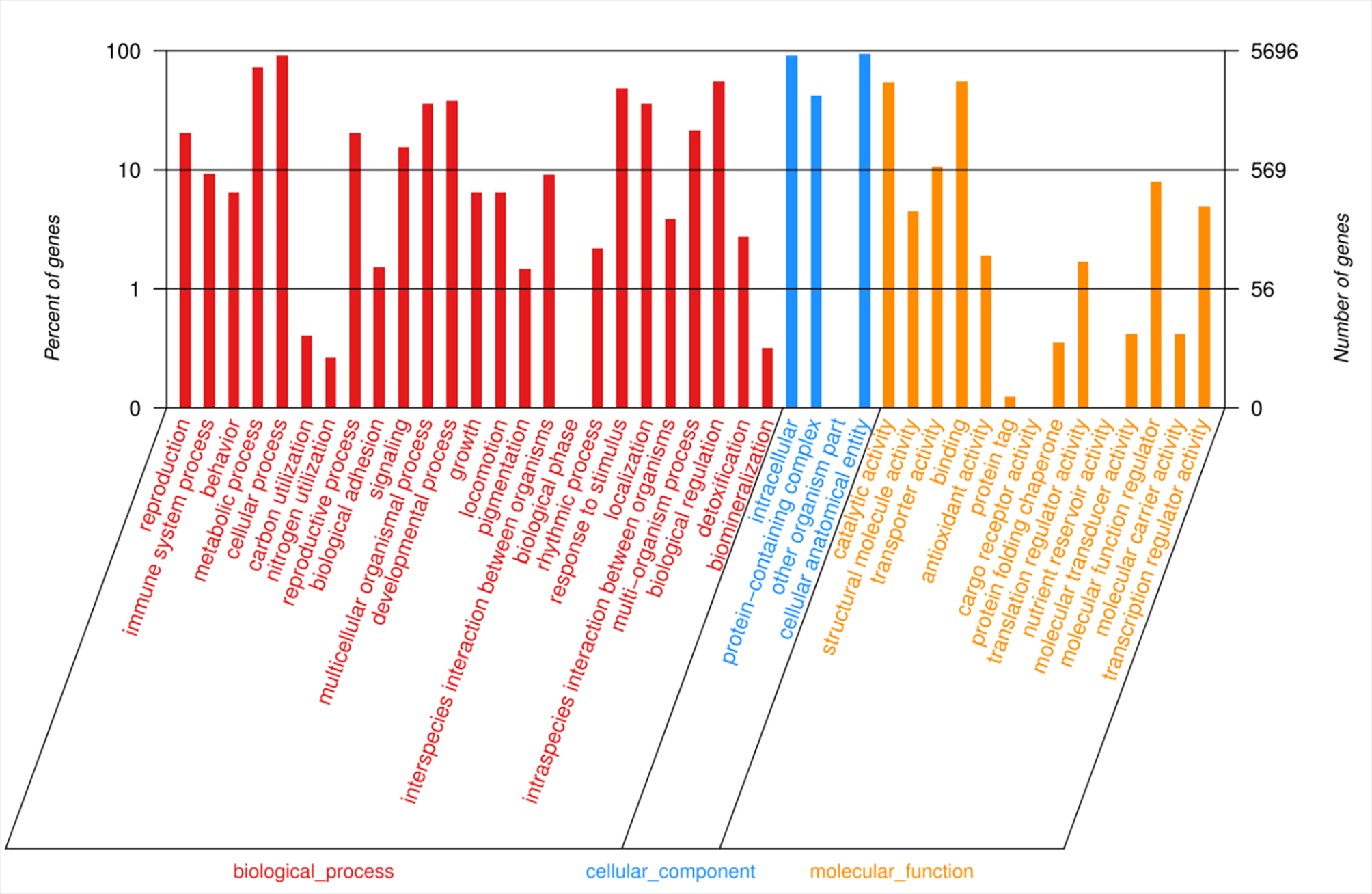

Supplement: Figure S2.tif [file KVIR_A_2590256_SM8831.tif]

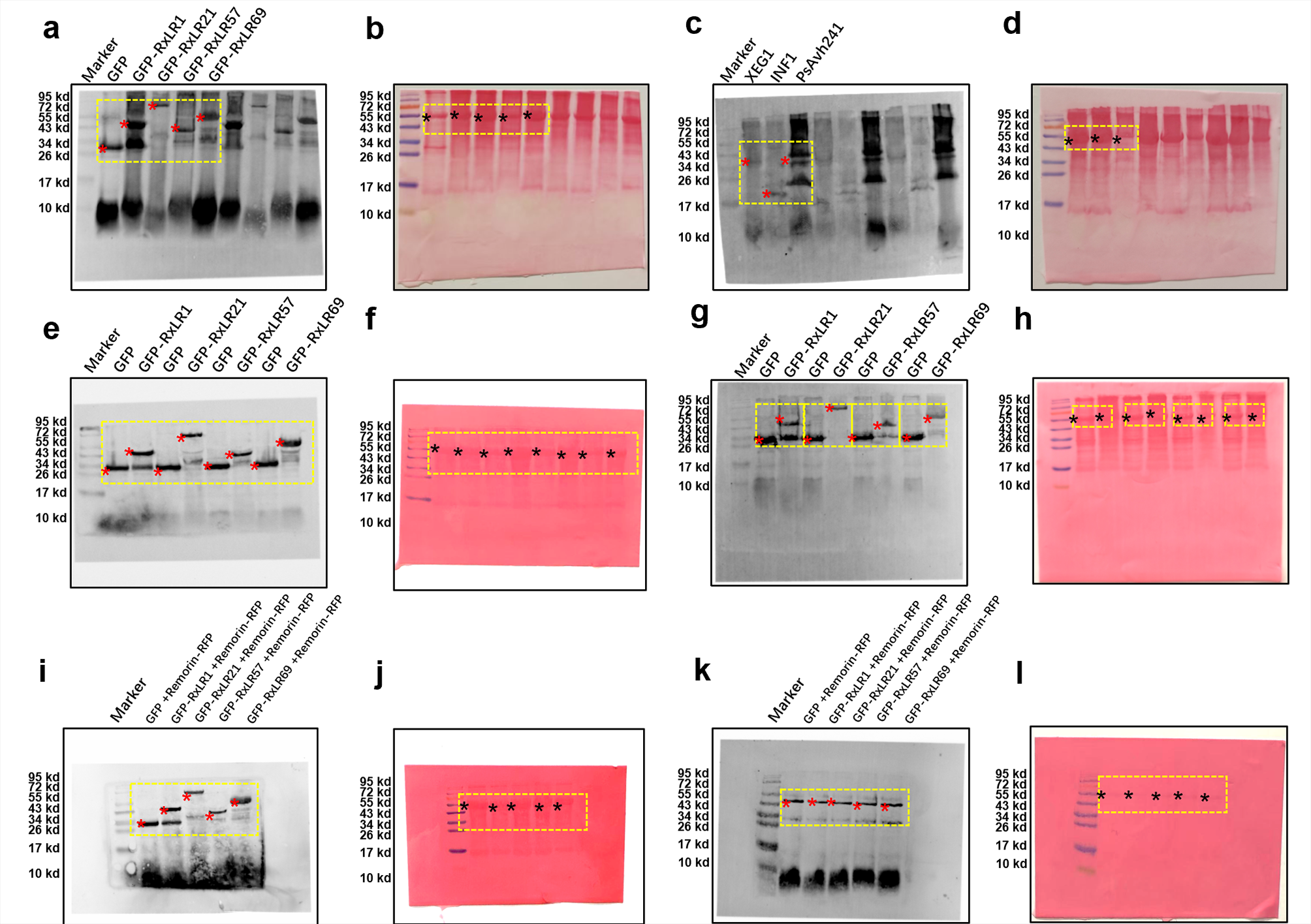

Supplement: Figure S6.tif [file KVIR_A_2590256_SM8829.tif]
