# Supplementary material for: Genome-wide identification and functional characterization of RxLR effectors in Phytophthora cinnamomi infecting Carya cathayensis Sarg
Source: Virulence. 2025 Nov 14;16(1):2590256. doi: 10.1080/21505594.2025.2590256 (PMC12629336; doi:10.1080/21505594.2025.2590256)
Supplement: Supplementary figures （clean version）.docx [file KVIR_A_2590256_SM8827.docx]

**Supporting information**


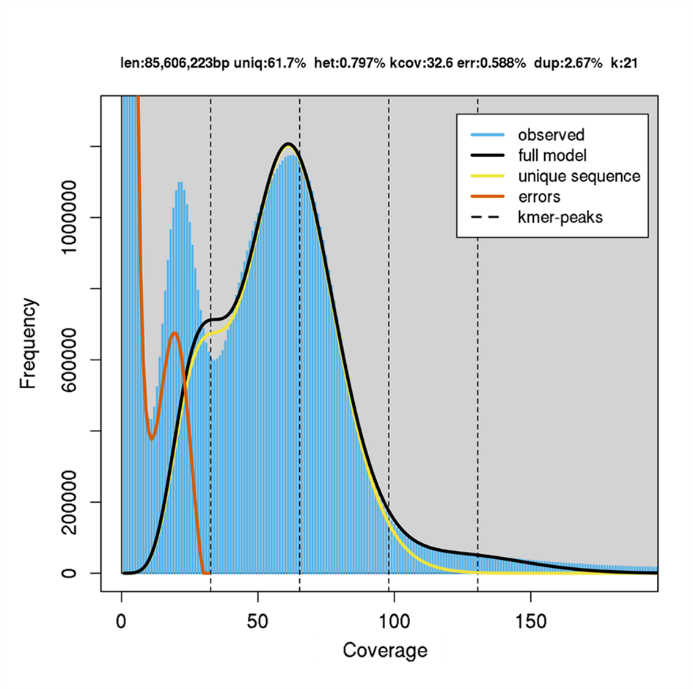


**Figure S1.** Distribution of K-mer frequencies in the genome. len: estimated genome size; uniq: proportion of non-repetitive sequences; het: heterozygosity; kcov: kmer coverage depth at heterozygous sites; err: proportion of kmers caused by sequencing errors; dup: proportion of duplicate sequences; k: kmer size used for evaluation.


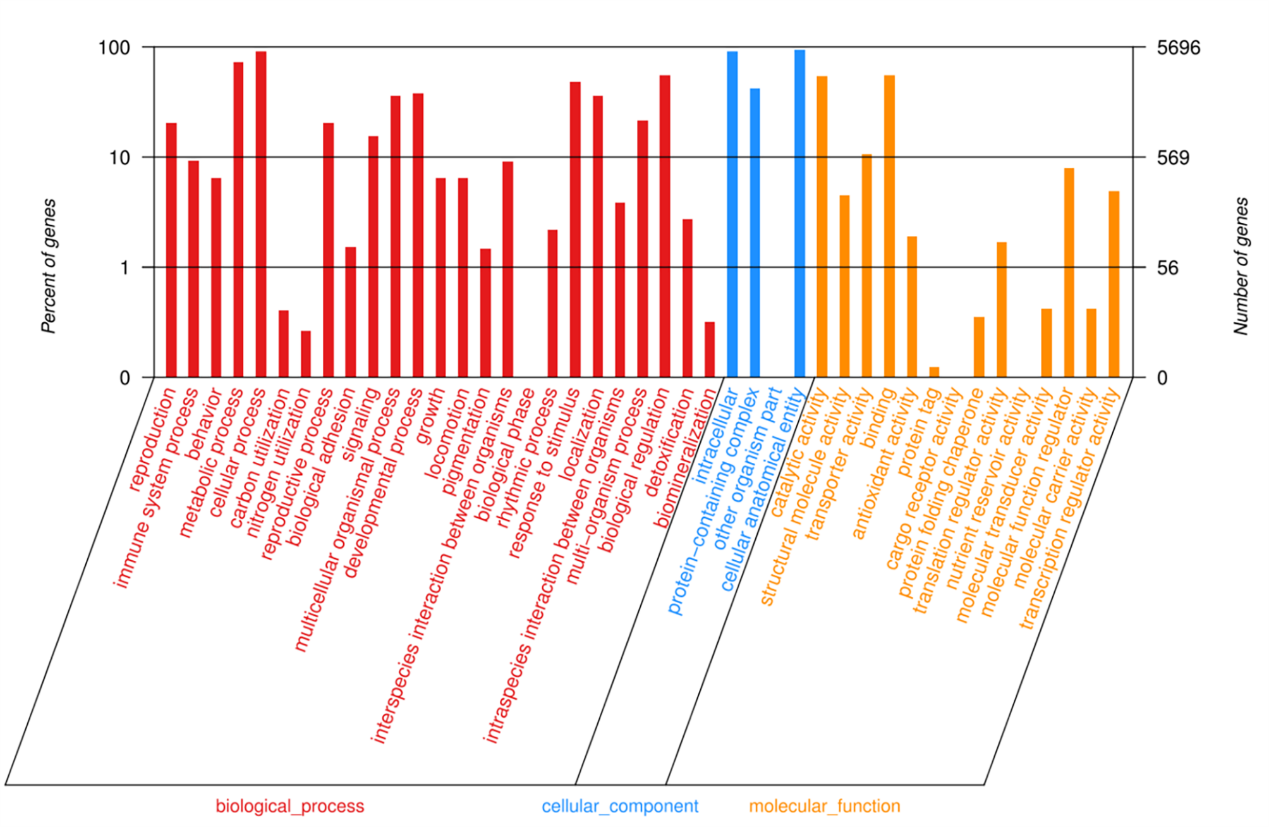


**Figure S2.** GO annotation of the genome of *P. cinnamomi*. Cellular component: the parts of a cell or its extracellular environment; Molecular function：the elemental activities of a gene product at the molecular level, such as binding or catalysis; Biological process: operations or sets of molecular events with a defined beginning and end, pertinent to the functioning of integrated living units: cells, tissues, organs, and organisms.


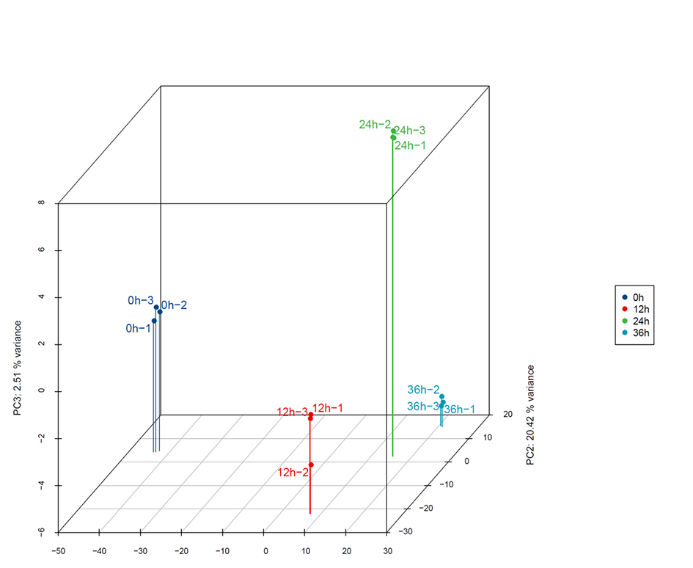


**Figure S3.** Results of principal component analysis among samples at different stages of infestation. The scatter plot displays the transcriptome profiles of samples collected at four different time points, with each point representing an individual biological replicate (n=3 per group).


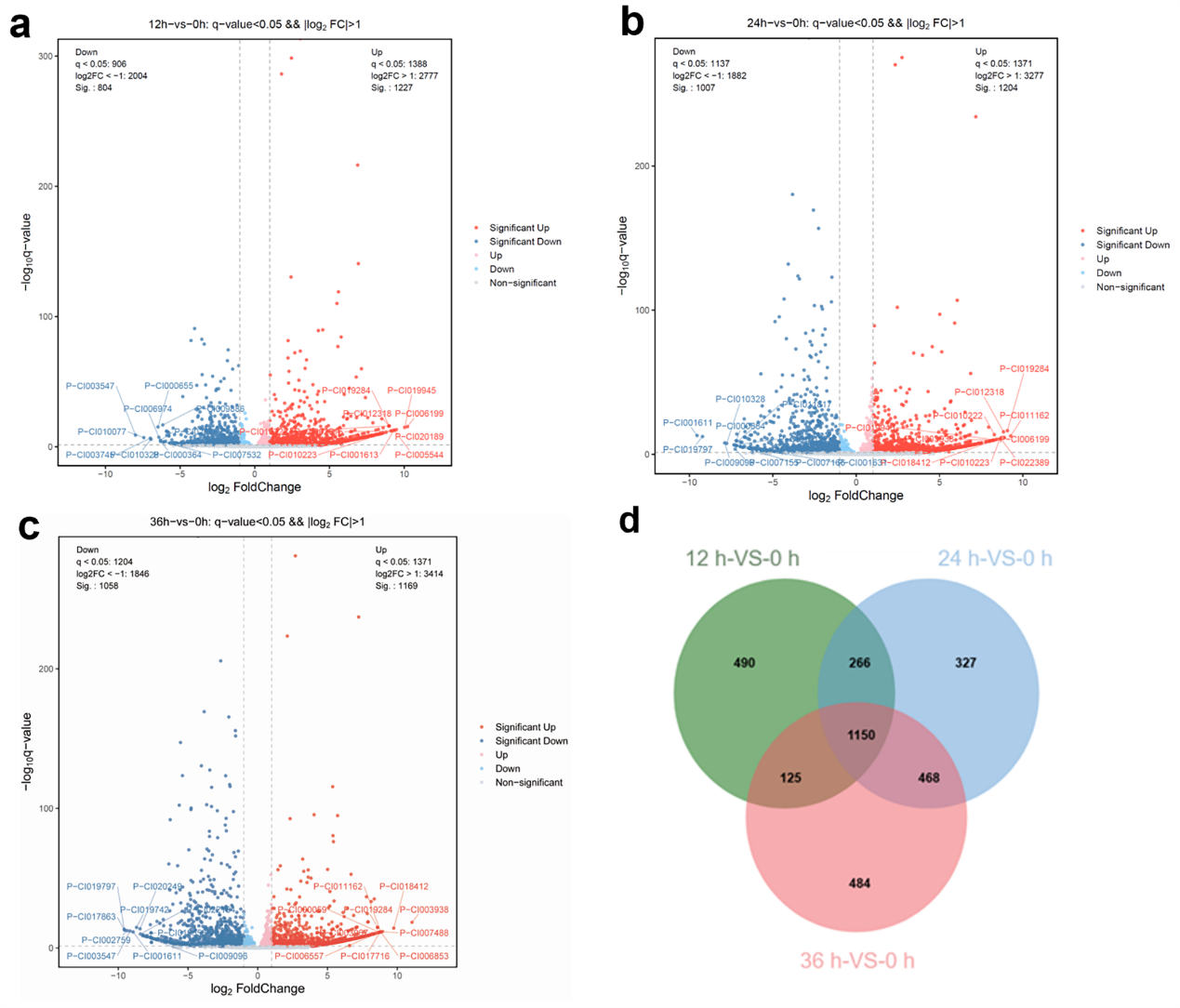


**Figure S4.** Analysis of differentially expressed genes in the early stage of infestation. (a-c) Differentially expressed genes at each stage of infection. Comparing the expression of various genes at each infection stage, using q<0.05 and │ log2FC │>1 as standards, a volcano map of differentially expressed genes between each infection stage and non-infection stage was obtained. Volcano plot was generated using the online tool (https://www.omicshare.com/tools/home/report/reportvolcano.html). (d) Cross of differentially expressed genes at each stage of infection. Venn was generated using the online tool (https://www.omicshare.com/tools/home/report/reportvenn.html).


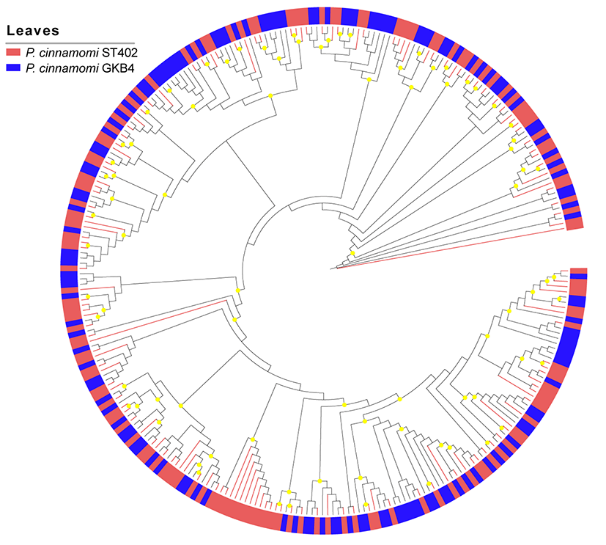


Figure S 5. Maximum-likelihood phylogenetic tree using RXLR-dEER motifs in RXLR effectors from five Phytophthora species. Yellow circles in the nodes indicate SH-aLRT values > 80%. Tree leaves are colored based on two P. cinnamomi strains. The branch containing the *P. cinnamomi* ST402 RxLR effectors was highlighted in red.


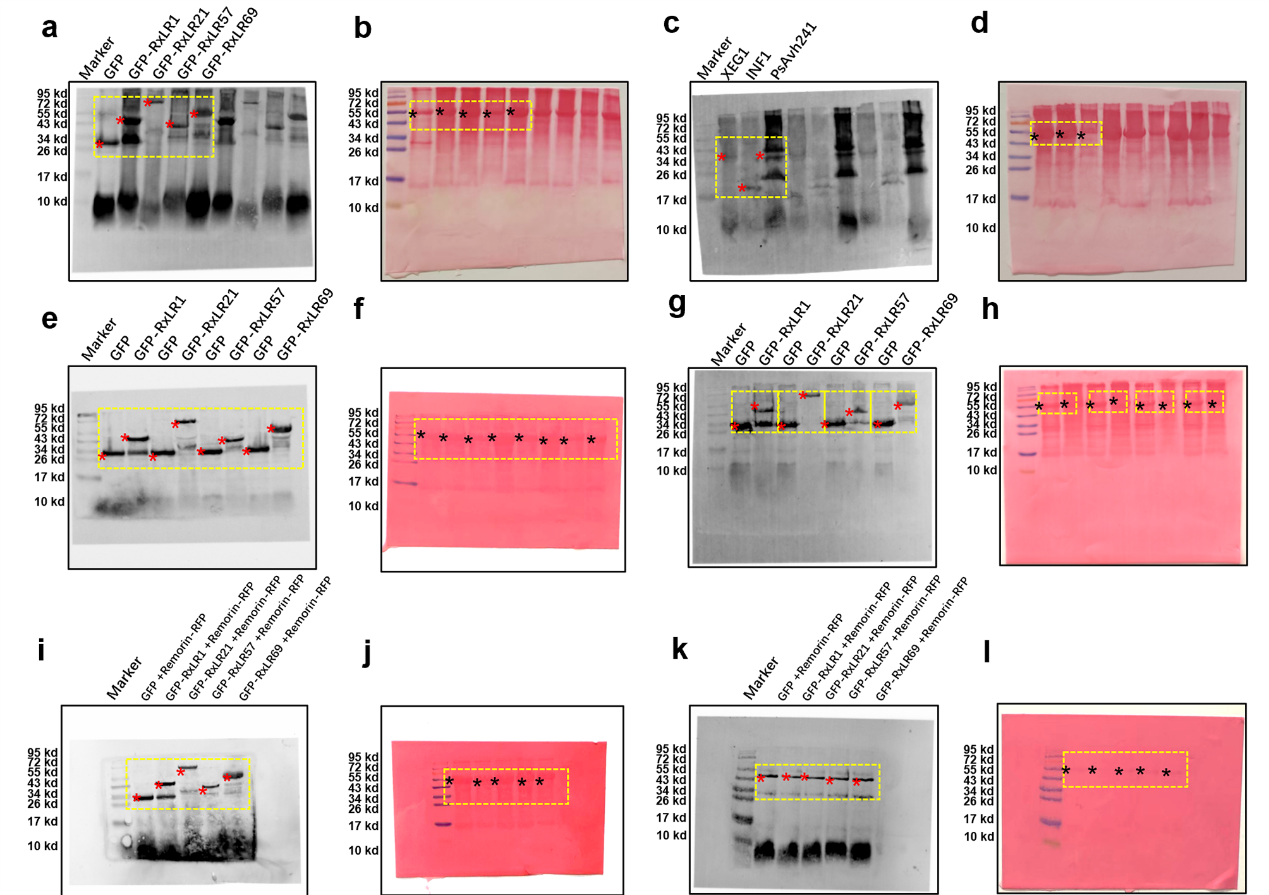


**Figure S6.** The individual uncropped, unprocessed image for each western blot. (a-b) Individual uncropped, unprocessed image for Figure 4b. (c-d) Individual uncropped, unprocessed image for Figure 4c. (e-f) Individual uncropped, unprocessed image for Figure 5. (g-h) Individual uncropped, unprocessed image for Figure 6e. (i-l) Individual uncropped, unprocessed image for Figure 7c. The Prestained Color Protein Marker used in western blotting (# P0068), bought from Beyotime, and the information of Prestained Color Protein Marker can be on the website: https://www.beyotime.com/product/P0068.htm
